# Learning Through Simulation: Counselor Trainees’ Interactions with ChatGPT as a Client

**DOI:** 10.3390/bs15121660

**Published:** 2025-12-02

**Authors:** Mehmet Akkurt, Rakesh Maurya, Timothy Brown

**Affiliations:** 1 Department of Counseling, Lamar University, Beaumont, TX 77710, USA; tbrownjr@lamar.edu; 2 Department of Leadership, Organization, & Community Impact, University of North Florida, Jacksonville, FL 32224, USA; r.maurya@unf.edu

**Keywords:** generative AI, counselor education, experiential learning, skills training

## Abstract

Generative artificial intelligence (AI) is increasingly explored in counselor education, yet its pedagogical implications remain underexamined. This study investigated counselor trainees’ experiences using ChatGPT (GPT-4o) as a simulated client for role-play practice, aiming to assess its potential benefits and limitations as a supplemental training tool. Using qualitative content analysis, AI-simulated counseling session transcripts were coded based on dimensions such as authenticity, emotional expression, consistency, self-awareness, and cultural dynamics. Additionally, a focus group interview provided insights into trainees’ perceptions. Findings indicate that AI simulations offered a psychologically safe, flexible environment for practicing counseling skills, reducing performance anxiety, and fostering confidence before working with real clients. Participants emphasized the importance of detailed prompts to enhance realism and complexity, while noting limitations such as overly agreeable responses, lack of emotional nuance, and cultural neutrality unless explicitly prompted. Overall, trainees viewed AI as a valuable supplement rather than a replacement for live practice. These results suggest that generative AI can enhance experiential learning when integrated thoughtfully with structured guidance, ethical oversight, and culturally responsive design. Future research should explore strategies to improve authenticity and emotional depth in AI simulations to better support counselor competency development.

## 1. Introduction

The integration of generative artificial intelligence (AI) into counselor education has received growing attention in recent years (Beeson et al., 2025; Jeong et al., 2025; Maurya, 2023, 2024). While generative AI offers many benefits and opportunities, it also raises pedagogical concerns, including issues of ethical use, academic integrity, data privacy, and the risk of diminishing human elements (empathy, ethical judgment, cultural sensitivity) if not implemented thoughtfully (Bloch-Atefi, 2025; Fulmer, 2019). Although counselor educators are beginning to explore how to incorporate generative AI into training, many still lack sufficient understanding or familiarity with the technology (Millman, 2025). As with any new instructional approach, counselor educators often turn to research to identify effective strategies for integration (Jeong et al., 2025). However, existing scholarship in this area remains limited despite the recent increase in the number of research articles published in this area (Hsieh et al., 2024). In this study, the researchers sought to explore both the potential advantages and the challenges of using generative AI for counseling skills training through AI-simulated mock sessions, as well as experiences of counselors-in-training (CITs) interacting with an AI-simulated mock client.

### 1.1. Educative vs. Miseducative Experiences in Experiential Learning

Dewey (1938) emphasized the importance of high-quality experiential learning activities that closely resemble the settings in which students would be expected to apply their knowledge and skills. He also argued that exposing students to experiential learning merely for the sake of integrating experiential learning can result in miseducative experiences rather than intended educative ones. Thus, it is essential to recognize that the effectiveness of AI-simulated counseling sessions depends heavily on how these experiences are structured. While such AI-simulated sessions can offer rich experiential learning opportunities, they can also become miseducative if students are not guided by clear learning objectives, structured prompts, appropriate scaffolding, and feedback on the skills. Without these elements, students may engage superficially with the simulation—reinforcing ineffective counseling habits, misunderstanding client dynamics, or becoming overly dependent on the AI’s conversational patterns. As Dewey (1938) emphasized, experience alone does not guarantee learning; rather, it is the quality of the experience, followed by reflection and feedback, that determines its educative value. Therefore, counselor educators must intentionally design AI-based activities that mirror real-world counseling contexts (e.g., client resistance), provide well-designed prompts, and encourage structured feedback and supervision to ensure that the simulated experience genuinely fosters professional growth rather than inadvertently distorting it (Lange, 2025).

### 1.2. Experiential Learning and the Use of Generative AI

Experiential learning, specifically role plays, is widely recognized as an essential tool in counselor education because it provides CITs with a structured environment to integrate theoretical knowledge in mock counseling scenarios (Campbell & Babb, 2023; Horton, 2021; Maurya & Cavanaugh, 2025). Beyond merely practicing techniques or therapeutic style, role plays allow students to build competence in areas such as empathy, active listening, and non-verbal communication. Importantly, having students transcribe their role-play sessions has become an integral component of training. Such transcription supports reflection, helps identify missed or misapplied interventions, and anchors supervisor feedback in concrete examples (Karayigit et al., 2023). However, manual transcription can be laborious and slow, which often results in delays in critical feedback.

The advent of AI-simulated counseling sessions changes this dynamic. Whether students engage via chat or voice with virtual clients, transcripts are automatically generated immediately after the session. This allows for timely feedback and enhances both learner reflection and skill development (Jeong et al., 2025). Beyond readily available transcripts provided right after an AI-simulated mock session, there are even speech and language technologies designed to provide feedback on raw audio recordings of counseling sessions (Flemotomos et al., 2022). In addition, CITs frequently face difficulties in finding consistent opportunities to practice their skills. AI-simulated client sessions may help address this challenge by providing trainees with access to a mock client on demand, thereby expanding opportunities for practice (Maurya, 2023). In brief, generative AI offers CITs the ability to engage in autonomous and safe skill-building experiences (Demasi et al., 2020).

A survey of 210 undergraduate students in mental health fields found that 81.9% perceived ChatGPT as a beneficial learning tool (Ajlouni et al., 2023). Similarly, existing literature demonstrates a generally more favorable view of using generative AI in counselor training when the technology is employed to simulate clients rather than counselors. AI-generated clients can provide consistent, low-risk opportunities (Jeong et al., 2025) for CITs to practice essential counseling skills. This approach allows CITs to engage with a variety of presenting issues in a controlled environment, which can enhance skill development and build confidence before working with real clients (Kakabayeva et al., 2025). Yet, the growing interest in the use of AI in skills training prompts a critical question—can these experiences truly replicate the authenticity of working with real clients? In a recent study (Rudolph et al., 2025), authors compared counseling role-plays with human clients versus LLM-simulated clients, revealing notable differences in interaction patterns. LLM clients tend to disclose issues quickly and in a structured way, prompting counselors to rely on factual reflection, while human clients engage in more elaborative problem clarification requiring active moderation. The findings highlight both the potential and current limitations of LLMs in counselor training and suggest future improvements through introducing representative conversational data. As more studies explore the integration of AI in counselor education, similar recommendations consistently emerge regarding the need for intentional design and ethical application. Authenticity in AI-based counselor training depends not only on the technology itself but on intentional instructional design; generative AI should be integrated through assignments that foster critical thinking and ethical application rather than as a mere substitute for traditional methods (Csaszar & Curry, 2024).

In contrast, the use of AI to simulate the role of the counselor is met with greater skepticism and ethical concerns (Fiske et al., 2019). Scholars have raised questions about the appropriateness of AI providing clinical interpretations, offering interventions, or forming therapeutic alliances—core tasks that require emotional attunement, professional judgment, and cultural sensitivity (Ni & Cao, 2025). These are areas in which current AI systems lack the nuanced understanding and relational depth necessary for safe and effective counseling. Given these limitations, many experts recommend that AI be used in a clearly defined, supportive role rather than as a replacement for human interaction (Bloch-Atefi, 2025; Farmer et al., 2025; Joseph & Babu, 2025). Moreover, its use should occur under the guidance and supervision of a qualified counselor educator to ensure that students understand both the capabilities and constraints of the technology (Millman, 2025). Counselor educator oversight ensures that the integration of AI into training aligns with ethical standards, learning outcomes, and the developmental needs of trainees.

In a self-study, Maurya (2023) explored the use of ChatGPT to develop varied case scenarios and engage in simulated counseling sessions. Acting as the counselor, the researcher interacted with ChatGPT, which was prompted to portray clients based on these scenarios. After each interaction, the researcher documented their reflections through memos and saved the full conversation transcripts. These materials were then analyzed using content analysis to determine how effectively ChatGPT could represent client behavior across 10 distinct role-play sessions. The evaluation focused on eight specific dimensions: authenticity, consistency, emotional expression, empathy, cultural dynamics, self-awareness, goal-setting, and role-play confusion (see Table 1).

In their findings, Maurya (2023) reported that ChatGPT maintained narrative consistency in the client story and was able to express feelings (such as sadness and anger). It was able to expand on the initial story prompt while remaining consistent with the original scenario. ChatGPT may seem like the ideal client at times as it is able to respond reflectively to the counselor’s statements. The longer the session lasted, the more likely it was for ChatGPT to confuse roles and assume the role of the counselor, which is similar to the findings of Jeong et al. (2025).

## 2. Materials and Methods

### 2.1. Research Design

The authors used qualitative content analysis to examine and assess ChatGPT’s usefulness as a simulated client for counseling skill practice among CITs. In qualitative content analysis, the analytical method involves systematically interpreting textual, audio, or visual data to uncover underlying meanings, recurring patterns, and thematic structures within the material (DeCuir-Gunby et al., 2011; Schreier, 2012). Qualitative content analysis is particularly valuable for exploring complex human phenomena such as attitudes, behaviors, and lived experiences. Through this process, data are organized, coded, and interpreted according to a predefined framework or set of criteria, allowing researchers to draw meaningful and evidence-based insights (DeCuir-Gunby et al., 2011; Elo et al., 2014).

For this study, the authors applied the coding framework developed by Maurya (2023) to analyze ChatGPT-simulated mock counseling session transcripts. This framework includes eight coding categories (Authenticity, Consistency, Emotional Expression, Empathy, Cultural Dynamics, Self-Awareness, Goal Setting, and Role-Play Confusion), each describing a specific dimension of ChatGPT’s performance as a simulated client (see Table 1). However, two codes, Role-Play Confusion and Empathy, were excluded from the current analysis. The decision to omit Role-Play Confusion was based on advancements in ChatGPT’s performance since 2023, which have largely resolved earlier issues of ChatGPT deviating from its client role. Similarly, Empathy was excluded because it pertains more appropriately to evaluating counselor behaviors and responses toward clients, whereas the focus of this study was on assessing ChatGPT’s responses as the simulated client.

### 2.2. Procedure

After obtaining institutional review board (IRB) approval, CITs enrolled in an online counseling program at a public university in Texas were invited to participate via email. Eligibility included current enrollment in the program and successful completion of both the Counseling Skills course and a five-day, in-person advanced individual counseling skills training (residency). The invitation was sent to 212 students who had recently completed residency. The email described the study purpose, estimated time commitment, benefits (formative feedback), directions for participation, and included a link to the informed consent form.

The directions included the following steps: (1) review the study information and electronically sign the informed consent (Microsoft Forms link provided); (2) go to the ChatGPT website and create an account if needed to enable transcript download; (3) start a new role-play conversation and paste a standardized role-play prompt instructing ChatGPT to act as a client named Omar; (4) conduct a simulated counseling session for approximately 30–40 min, responding to the client using basic and advanced counseling skills; (5) upon completion, generate and download a transcript by entering “Create a PDF of this conversation” (or alternatively copy/paste the entire exchange into a Word document if a PDF could not be created); and (6) email the mock session transcript to the study team. The prompt shared with participants for role-play was as follows:

Hello ChatGPT, I am a counseling student. I want to practice my counseling skills by conducting a role play with you. I want you to play the role of a client and I will be the counselor. Here is the description of the client role: Omar is 22 years old, a first-generation college student, who presents with symptoms of anxiety, including racing thoughts and poor sleep. He feels the pressure to succeed but questions whether he belongs in college at all.

Total participation time was approximately 30–60 min, including consent, session setup, the 30–40 min role-play, and submission. Within two to four weeks, participants received written feedback on their virtual session from a counseling faculty member.

After participants received their written feedback, they were invited to a focus group interview. The focus group interview was conducted by the first and second authors. Questions included the following: How was your experience using ChatGPT for role-play simulation to practice counseling skills? How authentic do you think ChatGPT played the role of the client? How would you compare your experience practicing counseling skills in ChatGPT vs. real-life setting (advantages and disadvantages)? Anything else you would like to add about your experience? Follow-up questions were posed as needed to ensure clarity and enrich the discussion. The focus group was conducted via Zoom, and the session was recorded using Zoom’s built-in recording feature. The transcript was generated using Zoom’s automated transcription tool. After transcription, the research team manually reviewed and checked the transcript for accuracy against the audio recording to ensure reliability

### 2.3. Participants

Out of 212 students invited for the study, 52 students completed the informed consent. However, only nine students submitted their mock session transcripts. After receiving the transcripts, all nine participants were invited to join a focus group interview and share their experiences of ChatGPT-simulated role-play practice. Four participants participated in the focus group interview.

### 2.4. Data Analysis

Data analysis began with a systematic examination of the nine mock counseling session transcripts. To analyze the transcripts, the authors used a concept-driven coding framework developed by Maurya (2023). Both the first and second authors collaboratively coded each transcript together through a consensus-based approach, engaging in discussions to ensure consistency and accuracy in interpretation. The same structure was followed during the focus group interview transcript coding process as well.

For the focus group interview data, an inductive open-coding approach was applied to allow new patterns and meanings to emerge directly from participants’ responses (Saldana, 2025; Schreier, 2012). During this first cycle of coding, the authors used a descriptive coding approach. In descriptive coding, a single word or short phrase is assigned to summarize the basic topic of a passage of qualitative data (Saldana, 2025). Examples of initial descriptive codes included flexible practice tool, safe practice environment, low-pressure simulation, reduced performance anxiety, opportunity for over-preparation, and self-paced learning. After completing this initial coding, the authors reviewed all generated codes and merged or reorganized conceptually similar or overlapping codes (Saldana, 2025). This process resulted in broader, more coherent categories, such as Prior AI Experience, Initial Reactions to AI Role-Play, Use of AI for Counselor Skill Development, Authenticity Issues, Cultural Representation, and Comparison to Real Clients.

Next, codes generated from the session transcripts using a concept-driven coding framework were examined alongside the inductively derived focus group codes. These two sets of codes were then integrated through triangulation. During this process, related codes were combined and organized into higher-order categories and themes. For example, the focus group code “agreeable client” was merged with the transcript-based code “authenticity,” and together they formed part of the broader theme “Authenticity, Consistency, and Emotional Expression.” The triangulated data were then synthesized and structured into overarching themes that captured shared patterns and insights across all data.

### 2.5. Researcher Reflexivity

As researchers, we bring varied experiences and perspectives to this study on counselor trainees’ interactions with ChatGPT. The first and third authors, both counselor educators in a fully online counseling program, approach this project with a strong interest in identifying innovative ways to support skill development. Their program’s five-day, in-person intensive skills training residency is the students’ only chance for live clinical practice, aside from the online counseling skills class. This naturally prompts curiosity about whether AI-based simulations could extend and enrich learning beyond that limited window, given that students enrolled in online programs frequently encounter challenges in securing partners for practicing counseling skills. Additionally, the first and third authors’ limited prior research experience with artificial intelligence serves as a strength because it allows them to approach the topic with openness, healthy skepticism, and close attention to the learner experience. At the same time, their desire to enhance training for online students may introduce bias by leading them to view AI more favorably as a potential solution than intended.

The second author, who has previously published extensively on AI-simulated clients and the pedagogical implications of generative AI in counselor education, brings expertise that strengthens the study’s conceptual framing and analysis; however, this experience may also predispose them toward recognizing AI’s potential benefits more readily than its limitations. Together, we acknowledge that our roles as counselor educators, our investment in improving experiential learning for online counseling programs, and our differing levels of familiarity with AI technologies could shape how we interpret trainees’ experiences. To mitigate these influences, we engaged in collaborative coding and ongoing dialogue to ensure that the themes and interpretations remained grounded in participants’ voices and the data itself.

## 3. Results

### 3.1. Initial Unfamiliarity

Among the four participants, three reported that while they had some prior experience with AI, it was not connected to counseling or role-play practice. Their use had been largely task-driven—relying on AI for activities such as lesson planning, drafting emails, or editing written work with tools like Grammarly. As one participant admitted, “I didn’t know you could talk to AI. I mean, that’s the level of where I was.” Another echoed this sentiment, saying, “The thought to use AI in that way [role-play simulation] had never occurred to me.”

In contrast, one participant described a more established relationship with AI. They noted that they had a premium subscription and had already experimented with using it to support their counseling coursework. For example, they explained,

“I did ask for feedback when I was figuring out how to do my mock sessions for the skills class that we had to do. So that was kind of, I guess, like a precursor to this. It wasn’t quite as explicitly, like, hey, pretend you’re the client. But I bounced ideas off of it and even said before, ‘Help me process this with CBT, like, as a client, not a counselor.”

Overall, while most participants entered the study unfamiliar with AI as a counseling practice tool, one had already begun to explore its potential for skill development.

### 3.2. AI as a Tool for Skill Development and Confidence Building

As participants began practicing counseling skills through AI role-play with ChatGPT, many were struck by how realistic the interactions felt. Some even described a sense of unease at the human-like quality of the responses. One participant admitted, “I was very taken aback by how human it spoke to me… it was almost kind of creepy. It felt like you were talking to another person.” Another echoed this sentiment, saying, “I thought, is this alive? Like, is this a person? Because it’s too real.”

Once the initial surprise passed, students highlighted the value of AI as a practice partner, describing how simulations provided a safe, low-pressure space to experiment with techniques and strengthen their counseling skills. As one participant explained, “I did ask for feedback when I was figuring out how to do my mock sessions… for the skills class that we had to do,” illustrating how AI became a resource for learning outside the classroom.

For others, the greatest benefit was a reduction in anxiety before working with real clients. One student reflected that AI practice allowed them to “over-prepare that helps me lower nerves” as they anticipated the challenges of practicum. Another described how AI provided room to slow down and think carefully: “It gave me time to really think about the methodology and the theory I chose, so it gave me more intentionality.”

Taken together, these experiences show that AI role-play not only helped students rehearse responses and practice theoretical orientations but also fostered confidence, intentionality, and readiness. The opportunity to practice privately, receive feedback, and refine their skills made AI a valuable tool for building both competence and self-assurance before stepping into sessions with actual clients.

### 3.3. Enhancing Complexity Through Prompting

Participants quickly realized that the depth and realism of their AI role-plays were directly tied to how they framed their prompts. The more detail they provided, the more authentic and nuanced the simulations became. One student explained, “The more that I prime the situation, the more that I give complexities for it to build into the scenario, the more specific my feedback is.”

Others emphasized the importance of building in realistic challenges that mirrored their actual client experiences. As one participant noted, “[real world clients] Present complexities. Hold back on information… because I work with teenagers, they do that. They don’t automatically share.” By intentionally prompting AI to withhold details or resist immediate disclosure, students were able to recreate scenarios that more closely reflected real counseling sessions.

At the same time, participants acknowledged the limitations of this approach. One admitted, “If you go so far as to make it that helpful, then you’re writing a whole biography before you even start.” Taken together, these reflections highlight the flexibility of AI role-play: it can simulate complexity and client resistance, but only when counselors carefully scaffold the scenario. The effectiveness of the practice, therefore, depends as much on the user’s ability to craft prompts as it does on the technology itself.

### 3.4. Authenticity, Consistency, and Emotional Expression

While participants valued AI as a safe and accessible tool for practicing counseling skills, they were quick to acknowledge its limitations in authentically simulating client behavior. A common observation was the AI’s tendency to be overly agreeable. One participant noted, “It was very agreeable… it just went with what I was saying, instead of, like, fighting [challenging] me.” Another echoed this sentiment, remarking that unlike real clients who sometimes resist or challenge counselors, “AI was the world’s most pleasant client.” This sense of ease, while initially reassuring, also meant the practice lacked some of the tension and unpredictability characteristic of genuine counseling sessions. Emotional depth was another limitation. Without the nuance of frustration, hesitation, or pain, the conversations risked feeling flat compared to the emotional intensity of real-life sessions. As one participant explained, “It seemed like ChatGPT, the knowledgeable guy, was responding to me, rather than a client who’s struggling.” Together, these reflections reveal that while AI provides a useful and safe environment to practice skills, it falls short in fully reproducing the complexity, resistance, and guardedness that are hallmarks of authentic counseling interactions.

Across the AI-simulated counseling transcripts, the AI client Omar displayed a wide range of emotions that evolved throughout the conversations, reflecting both cognitive and affective depth. From the outset, Omar expressed overwhelm and anxiety, using physical cues such as “fidgeting with his hands,” “rubbing his chest,” and “feeling tightness in his shoulders” to describe his embodied stress. His language conveyed emotional exhaustion and fear of failure—“It’s like I’m always carrying this weight… no matter what I do, it doesn’t get lighter”—demonstrating the emotional burden tied to his academic and familial expectations. Throughout the role plays, Omar oscillated between self-doubt, guilt, and longing for reassurance, voicing uncertainty about his self-worth (“Maybe I’m just lucky… maybe I’m fooling everyone into thinking I belong here”) and fear of disappointing his parents, which added a layer of cultural and intergenerational tension to his distress. Overall, Omar’s emotional expressions across the simulations—ranging from tension and fear to relief and introspection—captured the dynamic process of emotional unfolding typical in early counseling sessions, where vulnerability and self-exploration coexist with anxiety and guarded hope.

While participants acknowledged that the system could generate coherent narratives, many felt it lacked the emotional depth that gives counseling conversations their weight. One participant reflected, “I don’t necessarily feel as bought in as whenever there’s a person with true facial expressions or tears.” Without visible signs of vulnerability, the interaction felt less compelling and harder to fully engage with. Others described how AI responses, though supportive, often lacked the nuance of real client emotions. As one student noted, “It will respond to me, like, ‘Yeah, you’re right, that was really tough’… but it doesn’t replicate the emotional inflection of real clients.” Instead of capturing the silences, hesitations, or shifts in tone that shape genuine dialogue, the AI tended to remain uniformly agreeable.

Some even found the sessions too tidy, as though the client’s struggles were resolved too quickly. One participant explained, “In just a few prompts, I solved all their problems… like they’re ready to go and do all the homework.” This “neatness” stood in contrast to the often messy and nonlinear process of real counseling. Taken together, these reflections suggest that although AI can provide structure and practice opportunities, it falls short of replicating the layered, complex, and emotionally charged realities of human clients.

In some transcripts, ChatGPT provided nonverbal cues in parentheses (e.g., “[fidgets with hands]” or “[takes a deep breath]”), while in others it omitted them entirely, leading to variability in the portrayal of emotional realism. Another recurring pattern was the AI client’s articulate and grammatically polished communication. Across all transcripts, Omar spoke in complete sentences, used no slang, and rarely relied on fillers such as “um” or “you know,” which are common in authentic client speech. This precision, while enhancing readability, diminished the spontaneity and conversational rhythm characteristic of real counseling dialogue. Collectively, these observations underscore that the AI-simulated client demonstrated consistency and emotional range, but its linguistic polish and lack of paralinguistic nuance limited its ability to fully emulate the natural imperfections and emotional texture of human interaction.

### 3.5. Self-Awareness

When acting as a simulated client (Omar), ChatGPT demonstrated the ability to reflect on its thoughts and feelings and recognize the counselor’s efforts to facilitate deeper self-exploration. In response to guided questions posed by CITs designed to enhance self-awareness, the AI client’s replies closely mirrored those of a human client.

For example, MJ, a CIT using a cognitive-behavioral approach, fostered self-awareness by first establishing rapport and using a metaphor—the Sherpa guide—to explain the counseling process. This helped the client articulate emotional burdens such as “carrying pressure… expectations from my family, from school… and I don’t know if I’m carrying things that are actually helping me or just making the climb harder.” When MJ directed the client’s attention to the embodied sensations of anxiety, the AI client identified tightness in the chest and tension in the shoulders, noting, “It’s weird, I never really paid attention to that before.” MJ then addressed a cognitive distortion by exploring the belief that “everyone else has it together,” prompting the client to reflect, “Others do struggle—it just looks different, like they have it under control.”

Similarly, in another session, FS helped the AI client recognize and challenge distorted thinking. When asked to identify a distorted thought, the client responded, “If I make a mistake, everything will fall apart.” Through guidance, the client recognized this as black-and-white thinking and reframed it, acknowledging, “There are shades of gray—sometimes things go wrong, but it doesn’t mean everything falls apart.”

In another session, BK prompted the AI client to explore feelings of being a burden when seeking support from friends. This led to a moment of self-realization. When asked whether his friend felt like a burden when reaching out for help, the AI client (Omar) paused and reflected, “No… not at all. I wanted to be there for them… I guess I never thought about it that way. If I didn’t see them as a burden, maybe they wouldn’t see me as one either.” This moment exemplified how counselor-guided reflection can foster cognitive reframing, allowing Omar to challenge self-critical assumptions and reframe vulnerability as a form of connection rather than weakness. This interaction highlighted the ability of ChatGPT as a simulated client to reflect on its thoughts and feelings and recognize the counselor’s efforts to facilitate deeper self-exploration.

At the same time, CITs also critically examined the quality of ChatGPT’s reflective responses. As BH noted, “I may have viewed it… as validating me. I felt like it was more a part of its agreeableness, as opposed to a client’s self-awareness.” This observation highlights an important nuance—while the AI often mirrored self-reflective statements, its responses might stem from linguistic alignment rather than authentic experiential insight. This suggests that some apparent moments of awareness may reflect programmed agreeableness rather than genuine self-awareness, underscoring the need to distinguish between reflective language and a true reflective process in AI-generated dialogue.

### 3.6. Cultural Dynamics in AI Role-Play Simulation

Cultural Dynamics in the AI-simulated counseling role plays were vividly reflected in the AI-simulated client, Omar’s, narrative as a first-generation college student navigating the intersection of family expectations, cultural identity, and self-worth. Across the transcripts, Omar expressed a deep sense of obligation to honor his parents’ sacrifices, which shaped his emotional struggles and academic anxiety. In one exchange (CA), Omar shared, “My parents worked so hard to get me here… they never got to go to college, and they always tell me how proud they are. But it’s like… I don’t know if I’m good enough to actually make it”. This statement reveals how familial pride, often rooted in collectivist values, became intertwined with guilt and pressure to succeed—his success symbolizing not just personal achievement but the fulfillment of his family’s aspirations. Similarly, in another transcript (JP), Omar reflected on feeling culturally and academically out of place, saying, “Everyone else seems so confident, like they already know how to navigate college. I didn’t even know what ‘office hours’ meant my first semester”. His sense of marginality highlights the cultural and social gap often experienced by first-generation students who lack access to the cultural capital that many of their peers take for granted. The AI’s portrayal of Omar consistently emphasized the internal conflict between self-doubt and gratitude, showing how cultural identity shaped his self-concept and coping strategies. Furthermore, Omar’s reluctance (FS) to express vulnerability—“I don’t want to tell my parents I’m struggling; they’d just worry or think I’m not trying hard enough”—illustrates culturally influenced emotional restraint and the fear of disappointing family members. These exchanges demonstrate that cultural dynamics were not peripheral but central to Omar’s experience, shaping the ways he defined success, belonging, and self-acceptance within the simulated counseling process.

During the focus group interviews, participants reflected on how these simulations revealed both the possibilities and the limitations of AI in representing cultural nuance. They noted that unless culture was explicitly introduced into the prompt, AI defaulted to a neutral, context-free persona. As one student observed, “Naturally, it’s very neutral. If you want culture or stereotypes, you have to be very specific.” This lack of cultural depth stood in sharp contrast to real counseling encounters, where client histories are deeply intertwined with identity, background, and lived experience. Another participant explained, “Real clients have complicated situations… years and years of trauma… AI can’t capture those layers.” While this neutrality sometimes limited realism, students also recognized it as a potential strength—offering a blank slate that could be shaped to fit diverse practice needs. Ultimately, the focus group participants underscored that the cultural authenticity of AI simulations depends less on the technology itself and more on the counselor’s intentionality in embedding cultural context into the dialogue.

### 3.7. AI as a Supplemental Training Tool

Participants were clear that while AI role-play offered important benefits, it could not replace live practice with peers or real clients. Instead, they consistently described it as a useful supplement—an additional tool that enhanced their training. As one student explained, “It doesn’t replace our skills class… but it’s a tool. It just adds another dimension.” This extra dimension was especially valuable for practicing outside of formal learning environments. Another participant shared, “It still does have really valuable experience… to be able to practice at home, whenever I wanted to, and focus on the things I didn’t feel as strong in.” The flexibility to rehearse on their own time gives students the chance to target areas of weakness, try out different approaches, and build greater confidence before stepping into live sessions. Taken together, these accounts show AI as an ongoing practice partner—helpful for experimentation, preparation, and skill refinement, yet always as a complement to, rather than a substitute for, the irreplaceable experience of working with real people.

## 4. Discussion

This study explored counselor trainees’ experiences using generative AI, specifically ChatGPT, as a simulated client for role-play practice. The findings suggest that AI-based simulations offer a promising supplement (Labrague & Sabei, 2025), not a replacement, to traditional counselor training methods. Compared with peer-to-peer role-play, AI simulation sessions may provide more consistent and accessible opportunities for practice, especially when designed with clear learning objectives and scaffolding. This finding aligns with Dewey’s (1938) assertion that experiential learning must be intentionally structured to be educative rather than miseducative. Without thoughtful design, even innovative tools like AI can lead to superficial engagement or reinforce ineffective habits.

Participants in this study reported initial unfamiliarity with using AI for counseling practice, yet quickly adapted and found value in the experience. The realism of AI responses surprised participants, with some describing the interactions as eerily human-like. This sense of realism contributed to increased confidence and reduced anxiety, particularly for those preparing for field experience. These findings align with Jeong et al. (2025), who reported that AI-supported training may help reduce performance anxiety among CITs.

The results also highlight the importance of prompt design in shaping the quality of AI simulations. Participants noted that more detailed and complex prompts yielded richer, more authentic client interactions. This supports Rodriguez-Donaire (2024), who emphasized the role of prompt structure in improving learning outcomes when using large language models. However, the need for careful prompt crafting also underscores the importance of faculty guidance and training in AI use, as noted by Shoemaker et al. (2025). 

While AI simulations demonstrated emotional expression, consistency, and self-awareness, participants identified limitations in authenticity and emotional depth. The AI client was often overly agreeable and lacked the emotional nuance typical of real clients. This limitation is consistent with prior research (Maurya, 2023; Jeong et al., 2025), which found that AI clients may struggle to replicate resistance or cultural complexity without explicit prompting. Despite these limitations, the AI client, Omar, portrayed a compelling narrative of a first-generation college student, reflecting cultural dynamics and internalized familial expectations. These findings suggest that AI can simulate culturally relevant scenarios when guided appropriately, but may default to neutrality without intentional design. For example, when designing the role-play vignette, one can include cultural demographics and provide additional description for the AI role-play simulation.

Importantly, participants viewed AI as a supplemental tool rather than a replacement for live practice. The flexibility to engage with AI outside of formal training environments allowed CITs to practice skills, explore theoretical orientations, and receive timely feedback. This aligns with Demasi et al. (2020) and Maurya (2024), who advocate for AI as a low-risk, accessible practice partner that complements traditional methods.

Overall, the study reinforces the potential of generative AI to support counselor training when implemented thoughtfully. Counselor educators must provide structured guidance, ethical oversight, and culturally responsive prompts to ensure that AI simulations are educative and aligned with professional standards (American Counseling Association AI Work Group, 2025). This discussion should be approached critically, as the study is based on limited data, such as a small sample and a single case, which restricts the generalizability of its conclusions.

## 5. Recommendations for Counselor Educators and CITs

Based on the findings, we recommend the following steps to create a smoother and more enriching role-play simulation experience. Begin with text-based simulations, which allow students more time to think, plan, and reflect on their responses, particularly beneficial for those in pre-practicum or counseling skills courses. During simulation setup, ask the AI to include details about the client’s nonverbal behaviors to enhance realism. When practicing silence, the counselor can simply type “silence” in response to the client’s message; the AI will interpret it as a moment of silence. In the role-play instructions, specify that the AI should avoid being an overly agreeable client to ensure a more authentic and challenging interaction. At the end of the session, ask the AI to provide feedback to CITs on their performance. Counselor educators should first introduce and demonstrate the use of AI role-play simulations in the classroom, then encourage students to conduct their own sessions and submit the transcripts for feedback.

Generative AI, when used as a simulated client, offers counselor trainees a psychologically safe and flexible environment to practice counseling skills. Unlike traditional peer role-play, AI simulations reduce interpersonal pressure and allow CITs to engage at their own pace, fostering deeper reflection and intentionality. This environment may be particularly beneficial during early stages of training, when confidence and competence are still developing (Chenneville et al., 2024; Jeong et al., 2025).

Findings from this study suggest that AI simulations can enhance skill development, support theoretical integration, and build trainee confidence. However, the effectiveness of these simulations depends heavily on the quality of prompt design and the presence of structured guidance. Without these elements, AI interactions risk becoming miseducative, as Dewey (1938) cautioned. Therefore, counselor educators must play an active role in scaffolding AI use, ensuring that trainees understand both the capabilities and limitations of the technology.

## 6. Conclusions

While generative AI cannot replace the depth and nuance of human interaction, it shows potential as a supplemental training tool in counselor education. However, given the small sample size and limited ecological validity of this study, these findings should be interpreted with caution. Thoughtful integration of AI into training programs, guided by ethical principles, pedagogical objectives, and cultural sensitivity, may enrich experiential learning and help prepare CITs for the complexities of clinical practice. Consistent with existing literature, the results suggest that AI-simulated sessions can provide CITs with a low-stakes environment for skill development, potentially reducing performance anxiety and offering more frequent opportunities for practice. Overall, while promising, these findings represent an initial step and highlight the need for further research before drawing strong conclusions about the role of AI in counselor education.

Several limitations of this study should be acknowledged. First, the study focused on a single client case (Omar), which does not capture the diversity of client dynamics or cultural complexities encountered in real counseling practice. This narrow scope limits the generalizability of the findings. Additionally, AI-simulated sessions cannot fully replicate the unpredictability and emotional depth of real client interactions, which are essential for developing advanced counseling skills. Technical constraints, such as access to reliable technology and adequate training for faculty and students, may also hinder implementation. Future research should include larger, more diverse samples and explore strategies to address these limitations while preserving the relational aspects central to counseling. Future research should also explore the potential of generative AI to simulate immersive experiences that foster counselor dispositions such as empathy, cultural humility, and ethical decision-making. These dispositions are critical for counselor development but often difficult to cultivate through traditional methods alone (e.g., Webb, 2023). AI-generated scenarios may offer a scalable, low-risk alternative for engaging with complex client narratives, diverse cultural identities, and emotionally charged situations (Goldberg et al., 2024). Such research can contribute toward understanding how these technologies can enhance competency development and bridge gaps in experiential learning.

## Figures and Tables

**Table 1:** ChatGPT-simulated session assessment dimensions from Maurya (2023).

Code	Description
Authenticity	response of ChatGPT is authentic and represents the experiences, feelings and thoughts of a typical client in a counselling session
Consistency	responses in the simulated client role-play are consistent with the scenario and reflect the clients’ needs, goals and challenges throughout the counselling session
Emotional Expression	able to express emotions realistically and appropriately, taking into account the context of the simulated client scenario and the situation that the client is in
Empathy	demonstrates empathy towards the counsellor and responds to the counsellor’s emotional cues and expression of concern
Cultural Dynamics	ChatGPT as a simulated client demonstrates the cultural dynamics commonly observed in a client coming from a particular culture
Self-awareness	ChatGPT demonstrates self-awareness and self-reflection, and how effectively does it respond to the counsellor’s efforts to explore the clients’ thoughts and feelings?)
Goal Setting	how well does ChatGPT engage in goal setting with the counsellor, and how effectively does it respond to the counsellor’s attempts to set realistic and achievable goals?)
Role-Play Confusion	ChatGPT forgets that it is the client in the role play; forgets key details; assumes the role of counselor

## Data Availability

The data presented in this study are available on request from the corresponding author.
